# Novel Biodegradable Poly (Lactic Acid)/Wood Leachate Composites: Investigation of Antibacterial, Mechanical, Morphological, and Thermal Properties

**DOI:** 10.3390/polym14061227

**Published:** 2022-03-18

**Authors:** Mohammad Hassan Shahavi, Peyman Pouresmaeel Selakjani, Mohadese Niksefat Abatari, Petar Antov, Viktor Savov

**Affiliations:** 1 Faculty of Engineering Modern Technologies, Amol University of Special Modern Technologies (AUSMT), Amol 4615664616, Iran; p.pouresmaeel@ut.ac.ir (P.P.S.); m.n.sefat@alumni.ut.ac.ir (M.N.A.); 2 Faculty of Forest Industry, University of Forestry, 1797 Sofia, Bulgaria; victor_savov@ltu.bg

**Keywords:** antibacterial, biocomposite, biopolymers, mechanical properties, poly (lactic acid), wood leachate

## Abstract

This research aimed to investigate the effects of using wood leachate (WL) powder as a cost-effective filler added to novel poly (lactic acid) biocomposites and evaluate their mechanical, thermal, morphological, and antibacterial properties. Fourier transform infrared spectroscopy (FTIR), tensile test, Charpy impact test, Shore hardness, scanning electron microscope (SEM), differential scanning calorimetry (DSC), contact angle, and bacterial growth inhibition tests were employed to characterize the developed biocomposites. The SEM results indicated a proper filler dispersion in the polymer matrix. WL powder improved the hydrophobic nature in the adjusted sample’s contact angle experiment. Markedly, the results showed that the addition of WL filler improved the mechanical properties of the fabricated biocomposites. The thermal analysis determined the development in crystallization behavior and a decline in glass transition temperature (Tg) from 60.1 to 49.3 °C in 7% PLA-WL biocomposites. The PLA-WL biocomposites exhibited an antibacterial activity according to the inhibition zone for *Escherichia coli* bacteria. The developed novel PLA-WL composites can be effectively utilized in various value-added industrial applications as a sustainable and functional biopolymer material.

## 1. Introduction

In recent years, the enhanced environmental awareness, the increased need for sustainable and “green” materials, as well as the stringent legislative regulations related to waste management and cascading use of natural resources have forced many researchers to develop novel biodegradable polymers as a viable alternative to conventional polymers [1,2,3,4,5]. Despite the numerous advantages of biocomposites such as the potential to create a sustainable industry as well as enhancement in various properties such as durability, flexibility, high gloss, clarity, and tensile strength, there are certain drawbacks, such as deteriorated physical and mechanical properties, poor interface adhesion, brittleness, lower thermal resistance and water absorption, susceptibility to fungi and insect attacks, etc., limiting their wider application as functional materials [6,7,8,9]. Poly (lactic acid) (PLA) is one of the most suitable biodegradable polymers widely employed in many applications ranging from the biomedical field, e.g., in engineered drug delivery systems [10,11], tissue engineering (scaffolds) [12,13], and wound dressing [14,15] to food packaging and disposable plastic bags [16,17,18] due to its versatility, excellent processability, and biocompatibility. It is also widely used as a 3D printing feedstock for desktop fused filament fabrication 3D printers [19]. PLA is a biodegradable aliphatic semicrystalline polyester synthesized from lactic acid monomer (LA; 2-hydroxypropanoic acid), which is soluble in water and occurs in two enantiomeric forms, i.e., l-(+)-LA and d-(−)-LA. PLA represents a sustainable alternative to fossil-derived products, since it is derived from the fermentation of renewable agricultural biomass resources rich in carbohydrates, such as corn starch and sugar beets. Filler materials have been widely used in the polymer industries for economic reasons. Furthermore, it was reported that the fillers could also improve biocomposites’ mechanical and thermal properties [20,21,22,23,24]. Lignocellulosic waste and residues are among the most widely used fillers in polymeric composites. Using these natural materials will produce lighter products and lower the environmental footprint due to their biodegradability and lower cost. Another important benefit of using natural waste and by-products is significantly improving polymers’ mechanical properties [25,26,27].

**Figure 1:**
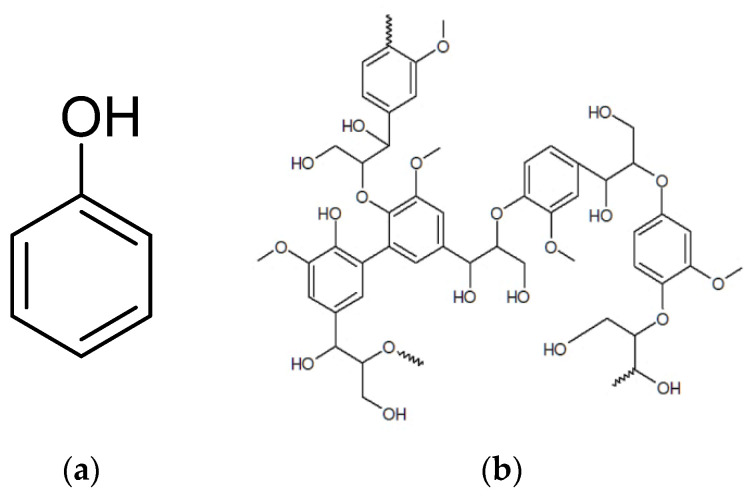
Structure of the main components of the wood leachate (WL) (**a**) phenol, and (**b**) lignin.

As in any wood-based industry, the medium-density fiberboard (MDF) production factories usually use wood as their feedstock [28]. In the process of MDF production and after wood chipping, the chips are washed with high-pressure steam. The waste produced by this process is a dark brown liquid called leachate. The wood leachate (WL) has a complex composition, but as mentioned in the literature, the leachate compounds are mostly phenol, tannins, lignin, and volatile fatty acids [29,30,31]. The structure of the main components present in the WL is shown in Figure 1.

The wood-based composite industry has developed rapidly during the last few decades, and its continued development is expected, e.g., the global demand for wood-based materials is projected to increase threefold between 2010 and 2050. In 2019, the annual global production of wood-based composites was estimated to be 357 million m^3^ [2]. This generates a massive amount of organic waste, i.e., from acid waste to aromatic leachates. In recent years, due to the increasing global environmental awareness, the need to decrease dependence on petroleum-based products, and new environmental regulations, great attention has been paid to the possibilities of developing biodegradable polymers from renewable, bio-based agricultural waste and residues [32,33,34]. Considering the great amounts of WL discharged by the wood-based industries, it is feasible to develop new methods for its efficient recycling and reuse in value-added applications. In addition, reusing WL waste solves waste management issues and has remarkable economic and environmental benefits. This acidic waste material could harm the environment and thus needs to be treated before discharge. Leachates may contain large quantities of organic pollutants such as ammonia (NH_3_), heavy metals, mineral salts, and phenol derivatives. Moreover, previous work has shown that this leachate can infiltrate and pollute the soil and the surrounding water, creating an environmental problem that needs to be addressed [35,36,37,38]. The WL’s main components, i.e., lignin, phenol, and tannin, can be used as a natural filler in polymer composites. The lignin in the WL could play a strong bioadhesive role in the polymeric blends, and the phenol and tannin could prevent bacterial growth on the composite surface [39,40].

This research aimed to evaluate the feasibility of using WL powder as an additive in PLA-based biocomposites and evaluate their exploitation properties. The chemical structure of the fabricated composites was characterized using Fourier transform infrared spectroscopy (FTIR). The effects of WL incorporation on the mechanical, thermal, and morphological properties of the PLA matrix were investigated using tensile tests, differential scanning calorimetry (DSC), and field emission-scanning electron microscopy (FE-SEM). Furthermore, the inhibition zone examination was used to detect the antimicrobial behavior of the developed PLA-WL biocomposites using *Escherichia coli (E. coli)* as the model microbe.

## 2. Materials and Methods

### 2.1. Materials

The PLA (CAS No: 26100-51-6) with a molecular weight of 80,000 g/mol, a specific gravity of 1.24 g/cm^3^, and a melting point of 155–170 °C was purchased from BIOKAS Ö5 (Chemiekas GmbH, Vienna, Austria). The raw WL (with 3% solids content) and fibers used in this work were supplied by the Arian Maryam Incorporation factory (AMI, Rasht, Iran) from the Arian Saeed Industrial Group. The lignin weight percentage of dry WL was determined as the acid-insoluble Klason lignin. The standard approach for determining lignin content is based on Klason’s method of hydrolysis of materials using 72% sulfuric acid (Merck 1.00748 98% diluted). Lignin is the substance remaining after hydrolysis in this step, and it is the insoluble residual extracted by filtration and measured by the gravimetric procedure [41,42]. The lignin content of dried WL was determined to be about 22%.

### 2.2. Biocomposite Preparation

To prepare the novel biocomposites, WL was vacuum dried at 100 °C in a vacuum oven (Memmert^®^ GmbH & Co. KG, Schwabach, Germany) at the first step. Then, the obtained solid material was grounded in powder using a lab-scale mill. The PLA was also vacuum dried at 70 °C to evaporate the moisture content. WL powder was dry blended with PLA and then melted compounded in an internal mixer (Brabender^®^ GmbH & Co. KG, Duisburg, Germany) at 180 °C for 15 min with the screw rotor speed of 50 rpm. The manufacturing parameters of the laboratory-fabricated PLA-WL biocomposites are given in Table 1. The prepared biocomposite blends were placed into a mold of 2 mm height, and the sheets were fabricated by a hot-pressing process in a laboratory press of 5 tons. The press temperature applied was 180 °C. The pressure value used was 1.4 MPa for 5 min [43].

### 2.3. Characterization

#### 2.3.1. Structural and Thermal Analysis

FTIR spectroscopy was employed to identify the composites’ functional groups. A Bruker Equinox 55LS 101 series instrument (Bruker, Karlsruhe, Germany) with a resolution of 4 cm^−1^ (50 scans on average) was used for this purpose. The Netzsch DSC Maia 200 F3 facilitated with a low-temperature accessory was employed for performing the DSC analysis. It was performed at a 20 °C/min heating rate at temperatures ranging from −20 to 280 °C through a nitrogen atmosphere. The stepwise specific heat increment midpoint was taken as the glass transition temperature (Tg). All DSC adjustments were made according to ASTM D3418 [44].

#### 2.3.2. Mechanical and Morphological Investigation

The tensile stress–strain experiments were accomplished using a Gotech Universal testing machine AI-7000-LA (Gotech Testing Machines, Inc., Taichung, Taiwan), following the ASTM D638 [45]. The examinations were carried out at room temperature (25 °C). The experiments were carried out at a 5 mm/min cross-head speed. At least five test specimens of any composition were considered for tensile tests. The ASTM D256 [46] standard was followed to perform the Charpy impact strength measurement of the developed composites. The notched test specimens (80 × 10 × 3.8 mm^3^) were tested using FRANK Baldwin-Model-BLI pendulum impact testing equipment (Frank Bacon, Warren, MI, USA). A 45 V-shaped notch was made in the center of the impact sample by razor notching equipment (CEAST). The notch tip radius was 0.25 mm, and the specimen depth remaining under the notch was 10.17 mm. For each composition, at least five samples were examined. Shore A sample hardness was measured using FRANK measuring instruments with ASTM D2240 [47]. Philips (Philips-X130) SEM equipment was employed for identifying the fracture surface of the prepared composite sheets in liquid N_2_. The SEM micrographs of the surface were obtained using the cold field emission mode on the surface of samples [48,49,50,51].

#### 2.3.3. Surface Hydrophobicity and Antibacterial Activity

The hydrophobicity of the laboratory-fabricated biocomposites was measured using a Kruss G10 contact angle measurement system (KRÜSS GmbH, Hamburg, Germany). The contact angle of the distilled water droplets was calculated on the composite surface [52].

The antimicrobial behavior of the developed composite materials was analyzed using *E. coli* bacteria. The *E. coli* bacteria was inserted into a liquid broth and cultivated under stirring of 120 rpm for 10 h at 37 °C in a shaking incubator. The resulting sample was diluted to around 100 (CFU)/mL using the broth media, and then, the diluted mixture was added to the agar medium. The PLA and PLA-WL samples were sterilized by autoclaving and placed on the plates with the agar’s surface in the tangent state and were incubated for 24 h at 37 °C. A comparative analysis of the biocomposites’ antimicrobial behavior measured the bacterial growth inhibition region (μm) [53,54].

## 3. Results and Discussion

### 3.1. Structural Characterization

**Figure 2:**
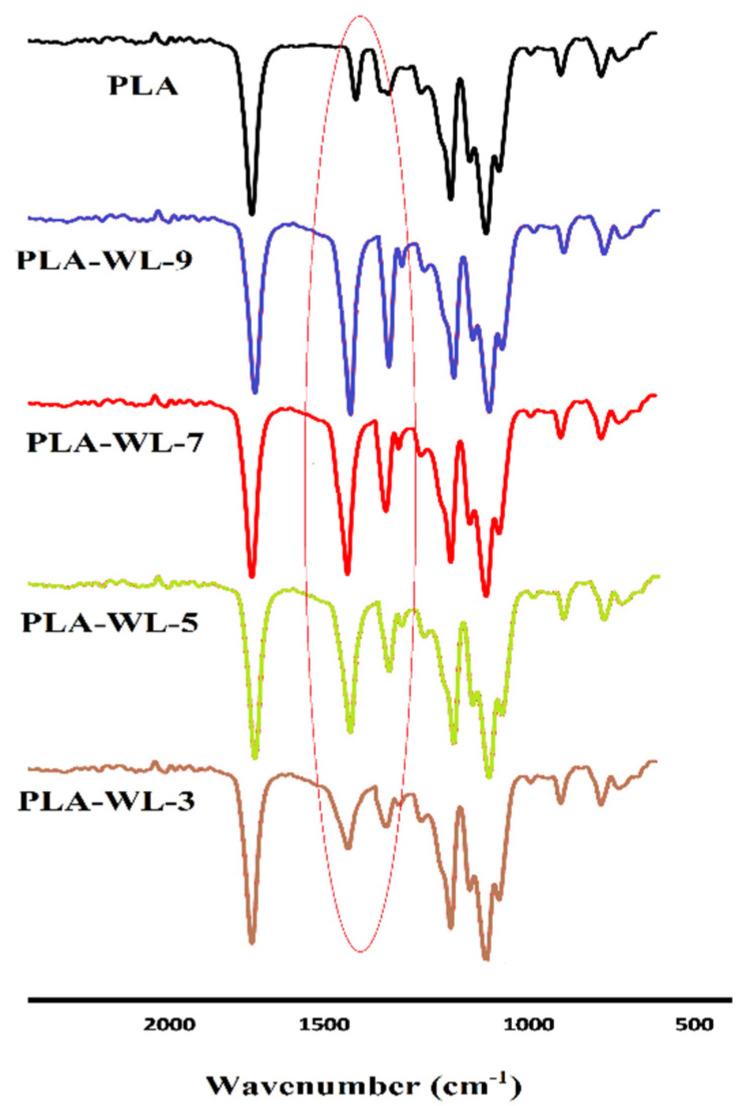
FTIR spectra of PLA and PLA-WL biocomposites.

The FTIR absorption peaks of the pure PLA and the PLA-WL biocomposites are shown in Figure 2. The C=O stretching absorption peaks related to PLA or ester groups were detected at 1750 cm^−1^. The C–O bonds in the PLA structure were visible at 1182 cm^−1^. The peak at 1363 cm^−1^ was related to PLA methyl groups and C–H bonds vibration. The C–C bonds stretching peak of the PLA chemical structure appeared at 836 cm^−1^.

After incorporating WL to the PLA matrix, the band near 1490 cm^−1^ was attributed to vibration of the phenolic rings, and the peaks at 1450, 1210, 1188 cm^−1^ for phenolic ring or phenolic hydroxyl groups vibrations [55,56] appeared in the spectrums of PLA-WL. That is proved by the WL’s phenolic structures (i.e., lignin, tannins, and phenol). The only difference between PLA-WL peaks are in their intensity that is related to the amount of filler and its compounds within the sample.

### 3.2. Mechanical Properties

The results obtained for the mechanical properties of the laboratory-made PLA-WL biocomposites, i.e., tensile strength, break elongation, and elastic modulus, are presented in Table 2.

**Figure 3:**
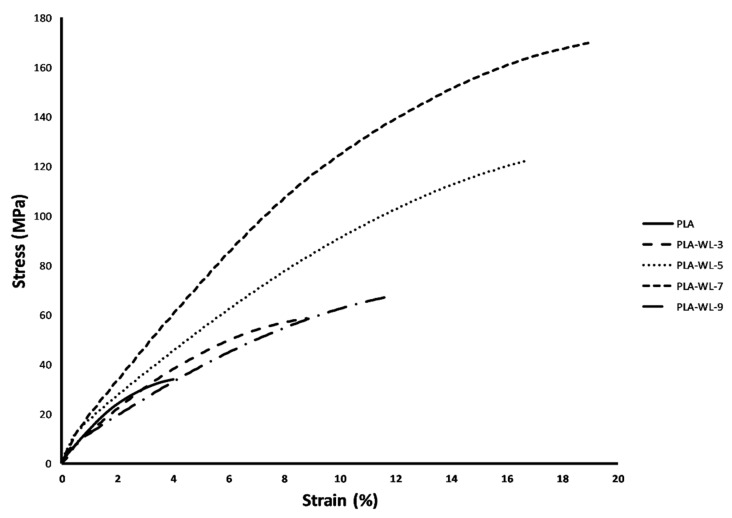
Stress–strain curves of tensile tests for neat PLA and PLA-WL biocomposites.

A graphical representation of the stress–strain curves of the developed PLA-WL biocomposites with different addition levels of WL as a filler is shown in Figure 3. Data are in correspondence to previously reported specifications for high molecular weight PLA [57]. The tensile strength of the PLA-WL blends was slightly increased by the filler loading increment (WL powder). The tensile strength was increased by about 400% at PLA-WL-7, which has a 7% concentration of the WL powder in its structure. The higher tensile strength of the filler-induced samples indicated the effect of lignin in the WL structure. The lignin has a paste-like impact within the polymer matrix that causes higher tensile strength values [58]. Lignin penetrates into the gaps of adjacent particles and intramolecular space of the PLA, and it improves the interfacial adhesion between WL filler and PLA matrix (physical adhesion) [59].

Markedly, the elongation at break was also increased in higher WL filler-loaded samples. This might be attributed to the plasticization effect of the lignin. Plasticization is described as the action of plasticizers on the matrix structure of materials, causing it to swell. Plasticizers reduce the T_g_ and melt viscosity by increasing the free volume by spacing polymer chains and increasing the mobility of chain segments. Lignin’s plasticization effect will also help to improve polymer matrix mobility via the effects of OH groups presents on lignin structure, which interacts with the polymer structure and leads to higher mobility as well as the processability and toughness of the resulting composite [60]. It was reported that high filler content increased the agglomeration probability [61], and the regions with stress concentration that requires less energy to crack will exist in this regard. The findings showed a significant decrease in tensile strength at 9% WL powder content. The localization of WL agglomerates between the PLA polymer chains in PLA-WL-9 resulted in decreased biocomposite break elongation. The steric hindrance of lignin, phenol, and tannin bonds in high WL powder concentrations leads to lower lock energy between the PLA chains.

The elastic modulus of the PLA-WL-3 specimen indicated a drop following WL’s addition due to lignin’s plasticization effect, referring to Table 2. The lignin mechanically bound the PLA chains in higher WL volumes, and the lignin’s paste activity exceeded its plasticization impact. Lignin could fit the polymer matrix, causing less mobility than the samples without or with a fewer percentage of WL powder filler [62].

During force loading in the testing machine, partial spaces were made, which barricaded the stress dissemination between the matrix and the filler. The degree of locking increased as the filler loading increased, raising the elastic modulus and stiffness. At the PLA-WL-9 sample, a sudden drop was observed related to the agglomeration of filler materials. The data relating to the Charpy impact strength of the fabricated films are presented in Table 2. The Charpy impact strength of the biocomposites was enhanced by the increment of the filler content from the results. Regular energy transfer in the matrix is substantial for impact persistence [63]. The highest impact resistant composition in this study was the PLA-WL-7 sample with an impact strength of 74.56 KJ/m^2^, and the factor has a growing trend by the addition of filler up to this composition. In dispersed phases addition into a polymer matrix, the dispersed phase’s good dispersion, filler particle sizes, and proper interfacial incorporation are the most critical factors determining the materials’ optimum performance. The blend’s impact strength drop with 9% WL powder was due to some unfavorable filler dispersion in the polymer matrix.

The composite’s hardness results are shown in Table 2. The hardness of the blended sheets increased with the higher filler content. It may be due to the WL powder dispersion and void minimization into the PLA matrix and intense physical engagement between the WL and PLA chains. However, the hardness increment value by filler addition has a slight trend, which is caused by dispersion of the WL within the structure and not just on the surface of the developed biocomposites. The highest hardness value of 92.3 was determined for the PLA-WL-7 sample. It can also be deduced that the filler increased the hardness of polymeric materials by filling the empty micro gaps between the polymer frameworks. Overall, the mechanical properties showed that 3–7% content of WL powder could be applied as filling material, leading to amelioration in the PLA composite’s mechanical properties, such as tensile strength, impact strength, and hardness. In addition, the induction of lignin, tannin, and phenol in low concentrations to the polymer blend can make it widely applicable in different industries according to the antibacterial effects of these materials, which are explained in the following section.

### 3.3. Thermal Behavior

**Figure 4:**
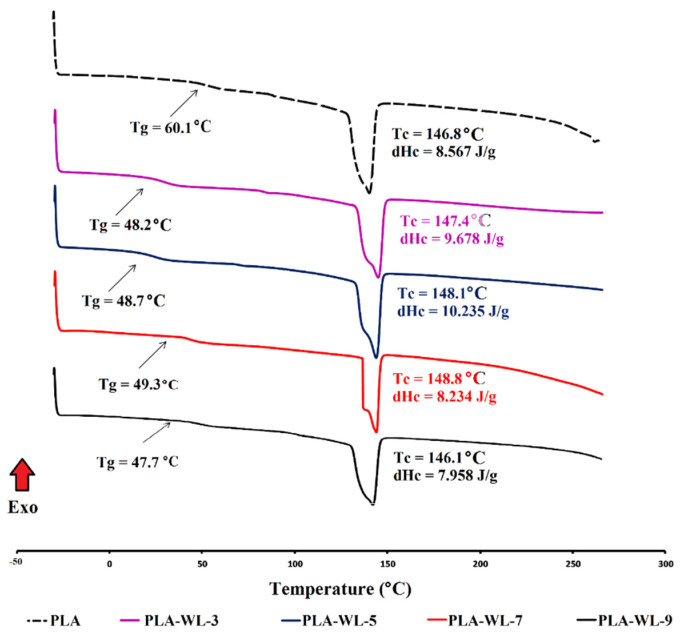
The DSC curves for PLA-WL biocomposites.

The DSC analysis was employed to investigate the effect of various filler (WL powder) loadings on the thermal characteristics of the developed PLA-WL biocomposites. Understanding the polymers’ crystallization behavior under dynamic conditions is considerable because most processing procedures perform in non-isothermal conditions [64,65]. Figure 4 illustrates the crystallization exotherms of the PLA-WL biocomposites. The parameters of the DSC curves for crystallization exotherms, such as the crystallization peak (Tc), glass transition temperature (Tg), and the crystallization enthalpy (dHc), were calculated from the under peak surface area.

The WL had a positive effect on the crystallization behavior of the PLA as the crystallization peak temperature was shifted to higher values after incorporating the WL filler. As expected, it also induced the crystallization enthalpy. The highest crystallization degree was determined for the PLA-WL sample. The higher crystalline structure of the PLA-WL-7 led to a high mechanically strengthened material [66,67], which complies with the results from mechanical testing. The plasticization effect of lignin leads to the free movement of the PLA chains, and as a result, the crystallinity of the modified composites became higher than that of neat PLA. On the other hand, high amounts of lignin and other phenolic compounds (in PLA-WL-9) present in the WL composition significantly reduced the polymer chains mobility caused by the steric hindrance impact associated with their aromatic composition.

Subsequently, the glass transition temperature was lowered by incorporating WL powder into the composite structure. The PLA had a Tg drop at 60.1 °C, and it is evident that WL lowers Tg as it makes the chains more mobile during the matrix due to its plasticization effect. According to the DSC results, the Tg has a positive trend by increasing the WL content in the laboratory-made biocomposites. This might be attributed to the chain locks occurring by increasing the filler amount. Lignin could hinder the polymer chains’ movement, resulting in greater Tg in higher WL filler ratios. Conversely, lignin could enhance the PLA chains mobility due to its plasticization effect, facilitating crystallization growth and improving crystallinity.

### 3.4. Morphology

**Figure 5:**
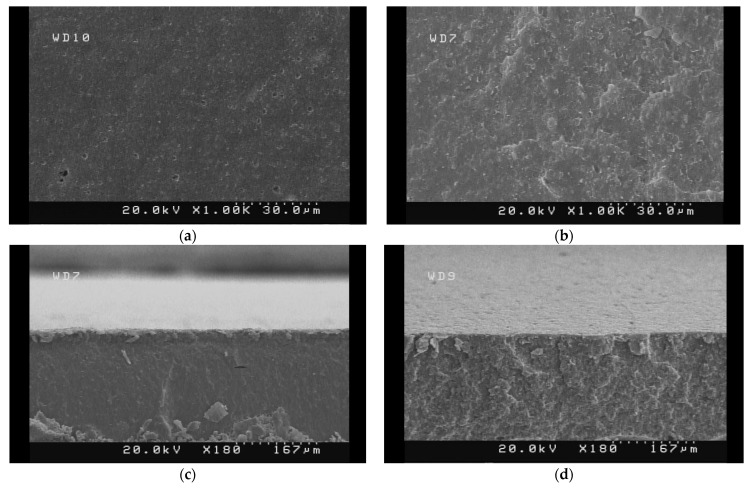

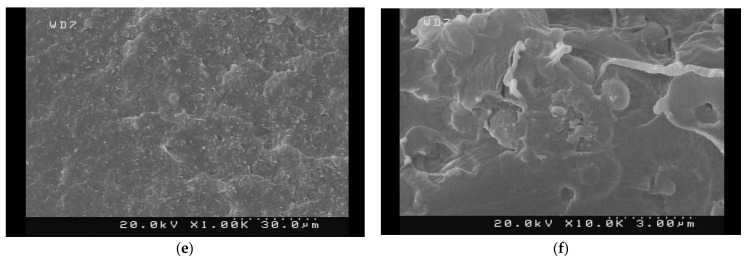
The SEM micrographs of the surface ((**a**) PLA and (**b**) PLA-WL-7 biocomposites), fracture surface ((**c**) PLA and (**d**) PLA-WL-7 biocomposites), and fracture surface of PLA-WL-9 in two different magnifications ((**e**) 1KX and (**f**) 10 KX).

The SEM technique was used to identify the biocomposites’ morphological modifications on the surface and cross-sections split in liquid N2. A graphical representation of the samples’ SEM images (PLA, PLA-WL-7, and PLA-WL-9 biocomposites) is presented in Figure 5.

The PLA had a smooth surface with some voids related to the humidity vaporization from the surface during the hot pressing process. The uniform structure was detected for PLA in both surface and cross-section images. The WL powder’s presence in the structure of the PLA-WL-7 biocomposite was completely observable from changes in the morphology in the SEM micrographs of this sample. Different phases were detectable, and WL powder was well dispersed between the PLA matrixes. No micro-domains were observed in the developed PLA-WL composites. The homogeneous surface resulted from the obtained good dispersion of WL in the PLA matrix during the process, confirming the composites’ enhanced mechanical performance containing 7% WL. The optimal mechanical and thermal characteristics were determined for the PLA-WL-7 sample. These improvements were proved by illustrating WL proper dispersion in the polymer matrix. A powerful filler and matrix engagement due to the lignin’s paste impact was evident in the images. The SEM micrographs of the fracture surface for the PLA-WL-9 sample are illustrated in Figure 5e,f. Some agglomerations of WL are detectable within the image. The results correspond with the mechanical testing results due to the reduced mechanical strength of this sample compared with the PLA-WL-7 composite.

### 3.5. Contact Angle

**Figure 6:**
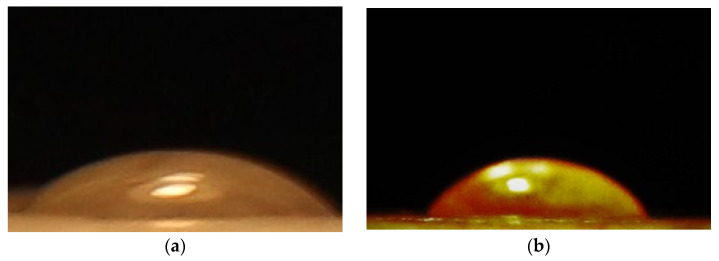
The contact angle images for fabricated biocomposites ((**a**) PLA and (**b**) PLA-WL-7).

The developed biocomposites’ contact angles determined the surface wettability (Figure 6). The contact angles increased from about 46° to 66° when the WL was incorporated into the PLA matrix at 7 wt % concentration. It was observed that the addition of phenolic compounds which are presented in the WL enhanced the hydrophobicity of the composite surface. The reason for this hydrophobicity is the emplacement of the WL with hydrophobic phenolic functional groups in the micro spaces of the PLA matrix. The data achieved from the contact angle tests agree with the antibacterial activity test, which is reported in the next section. This study may serve as a stepping stone for future investigations to solve some of the critical problems of bioplastic industries by preventing moisture diffusion and inducing the antibacterial activity into a biodegradable packaging material, i.e., PLA.

### 3.6. Antibacterial Behavior

**Figure 7:**
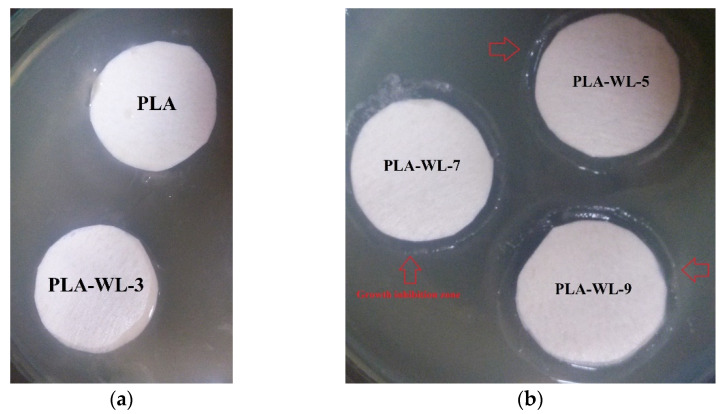
The antibacterial activity of the prepared biocomposites ((**a**) PLA and PLA-WL-3 and (**b**) PLA-WL-5, PLA-WL-7, and PLA-WL-9) against *E. coli*.

The materials’ antibacterial activity is associated with their physical and chemical properties such as the functional groups, dispersion of antibacterial agents, and surface roughness. It is well known that bacteria tend to be cultured on hydrophilic surfaces, and thus, hydrophobic surfaces may inhibit bacterial growth [68,69,70]. For examining the antibacterial characteristics of PLA-WL biocomposites, the association of the *E. coli* bacteria with neat PLA and PLA-WL was mentioned. Figure 7 indicates composites’ behavior in the forms of cytotoxic effects. The density of the bacterial growth around the neat PLA indicates that there is no antimicrobial activity in the neat PLA structure. WL powder insertion into the PLA matrix structure led to a lower microbe density across the test sheet, suggesting an inhibitory effect. The results illustrated that the PLA-WL-3 has no bacterial growth inhibition, unlike the neat PLA sample. On the other hand, PLA-WL-5, PLA-WL-7 and PLA-WL-9 have shown growth inhibition zones, and this activity is more indicatable in PLA-WL-9. This result proved the claim of the antibacterial effect of WL on *E. coli* growth. The expressed antimicrobial behavior of the PLA-WL composites proved their potential use in various packaging applications to inhibit bacterial growth. The authors postulate that phenolic compounds in the WL structure caused the PLA-WL composite’s antibacterial properties.

## 4. Conclusions

The results indicated that the WL powder filler induction in the PLA matrix could enhance the polymer’s mechanical characteristics. WL significantly improved all of the PLA’s mechanical properties, such as tensile strength, elongation at break, and hardness. The addition of WL resulted in an enhanced Charpy impact strength of the biocomposites. The PLA’s thermal stability and crystallization behavior were also improved with the addition of WL as a filler. The surface hydrophobicity of the PLA-WL biocomposites was improved compared to the neat PLA sample. The antibacterial activity is another benefit that the WL added to the PLA. In bio-industries, preventing moisture diffusion and bacterial growth are very significant challenges. The induction of these properties to the PLA as a biopolymer could make the WL suitable as a filler for various industrial applications.

## Figures and Tables

**Table 1:** Manufacturing parameters of PLA-WL biocomposites produced in this work.

Sample	PLA (%)	WL (%)	Mixing Temp (°C)	Mixing Time (min)	Sheet Thickness (mm)
PLA	100	0	180	15	3.4
PLA-WL-3	97	3	180	15	3.6
PLA-WL-5	95	5	180	15	3.7
PLA-WL-7	93	7	180	15	3.5
PLA-WL-9	91	9	180	15	3.9

**Table 2:** The mechanical characteristics of the fabricated PLA-WL biocomposites.

Sample	Tensile Strength (MPa)	Elongation at Break (%)	Elastic Modulus (MPa)	Charpy Impact Strength (KJ/m^2^)	Shore A Hardness
PLA	33.95	4.00	1837	38.62	89.21
PLA-WL-3	58.65	8.80	1553	46.21	91.32
PLA-WL-5	121.75	16.73	1817	62.47	91.85
PLA-WL-7	169.75	18.93	1942	74.56	92.30
PLA-WL-9	66.95	11.71	1427	61.84	86.21

## Data Availability

Not applicable.
